# Semiparametric Mixed Models for Medical Monitoring Data: An Overview

**DOI:** 10.4172/2155-6180.1000234

**Published:** 2015-06-26

**Authors:** RD Szczesniak, D Li, SA Raouf

**Affiliations:** 1 Division of Biostatistics and Epidemiology, Cincinnati Children’s Hospital Medical Center, Cincinnati, USA; 2 Department of Mathematical Sciences, University of Cincinnati, Cincinnati, USA; 3 Division of Pulmonary Medicine, Cincinnati Children’s Hospital Medical Center, Cincinnati, USA

**Keywords:** Ambulatory blood pressure, Covariance models, Linear mixed models, Longitudinal data, Functional data analysis, Nonparametric regression, Obstructive sleep apnea, Penalized splines, Semiparametric regression, Serial correlation

## Abstract

The potential to characterize nonlinear progression over time is now possible in many health conditions due to advancements in medical monitoring and more frequent data collection. It is often of interest to investigate differences between experimental groups in a study or identify the onset of rapid changes in the response of interest using medical monitoring data; however, analytic challenges emerge. We review semiparametric mixed-modeling extensions that accommodate medical monitoring data. Throughout the review, we illustrate these extensions to the semiparametric mixed-model framework with an application to prospective clinical data obtained from 24-hour ambulatory blood pressure monitoring, where it is of interest to compare blood pressure patterns from children with obstructive sleep apnea to those arising from healthy controls.

## Introduction

Medical monitoring plays a major role in modern clinical studies, ranging from portable actigraphy devices worn by individual subjects [1] to at-home reporting of biologic markers [2]. Simultaneously, there is growing interest in the prognostic utility of medical monitoring for clinical care of patients with chronic diseases and disorders [3]. In addition to static variables collected on individuals, a longitudinal sequence of measurements is collected. Data arising from medical monitoring can have complex, time-related dependencies [4]. The analysis of medical monitoring data focuses on the evaluation (and possibly prediction) of the individual’s time course.

Both early origins and more recent approaches to longitudinal data analysis share the challenge of fitting nonlinear trends to observed data over time. Notable examples include the use of the linear mixed model (LMM) to analyze longitudinal data [5], and more recent work has involved adaptive curve fitting [6]. Semiparametric regression, a compromise of linear and nonlinear regression modeling, was thoroughly described by Ruppert, Wand and Carroll [7]. This methodology typically refers to specifying nonparametric components for effects that vary over a continuum, such as time or space, and parametric effects for the remaining model structure (e.g. covariate effects and subject-related random effects or measurement error terms).

In this paper, we focus on the application of a particular type of semiparametric regressions, known as semiparametric mixed models, to medical monitoring data and interpretations that can be made using these models when it is of interest to detect rapid changes in the mean response function and compare experimental groups. Although semiparametric mixed modeling has gained popularity in many of the sciences, its use in medical monitoring is relatively new. We review the general model framework and inference, provide detail on practical applications, illustrate more recent approaches with a case study and describe future work that may further increase the clinical utility of these models.

## General Semiparametric Mixed Model Framework

We present a general formulation for the semiparametric mixed model that includes a nonparametric component for the time-varying effect and parametric component(s) for any other effect(s) in the model consistent with established formulations [7]. Suppose we observe data on individuals over time, which yields subject-specific collections of data, often known as profiles. Consider medical monitoring data that consists of a sequence generated by a longitudinal process ${\left\{Y_{ij}\right\}}_{j=1}^{n_{i}}$, which is specific to each individual (*i*=*1,*… *n*) at points t_ij_(*j*=*1,*… *n_i_*) along the continuum of time. If the process exhibits sharp changes or nonlinear trends over time, it may be difficult to parametrically model these effects, even with polynomial regression [8]. As an alternative, we can specify such effects as functional data [9]. A semiparametric mixed model for this process can be expressed as:

(1) $$Y_{ij}=X_{ij}^{T}\theta +f\left(t_{ij}\right)+Z_{ij}^{T}b_{i}+\varepsilon_{ij},$$

Where parametric effects are represented for traditional (linear) covariates X_ij_ and p × 1 parameter vector θ; f(.) is a nonparametric smooth function evaluated at t_ij_; z_ij_ and b_i_ correspond to the design matrix and subject-specific q_i_×1 parameter vectors for random effects. The term ε_ij_ comprises serial correlation and the measurement of residual error corresponding to the observation at time t_ij_.

It is well known that accounting for intra- and inter-subject variability is a pervasive issue in longitudinal data analysis. As an example using Equation (1), we could assume that ε_ij_ follows an independent, identically-distributed Gaussian distribution, denoted as $\varepsilon_{ij}\sim N\left(0,\sigma_{\overset{ˋ}{o}}^{2}\right)$, and incorporate subject-specific variation by specifying random intercepts, U_i_, where *U_i_* ⊥ *ε_ij_* for all i and j. It is not uncommon with medical monitoring data, however, to observe sequences on a given subject at irregularly spaced time points and at different time points between subjects. An alternative covariance structure, perhaps more appropriate for such data, is:

(2) $$\varepsilon_{i}\left(t_{ij}\right)=\delta_{i}\left(t_{ij}\right)+\omega_{ij}$$

Here *δ_i_*(*t_ij_*) is a correlation function that changes over time and is assumed to follow a stationary Gaussian process with mean 0 and variance $\sigma_{\delta }^{2}$;*ω_ij_* is the “white noise” component, where $\omega_{ij}\sim N\left(0,\;\sigma_{\omega }^{2}\right)$. Replacing ε_ij_ in Equation (1) with the more general serial correlation structure in Equation (2) yields the semiparametric stochastic mixed model presented by Zhang and colleagues [10].

In settings with medical monitoring data, *δ_i_*(*t_ij_*) could be specified as exponential decay. The correlation function, *Corr*(*δ_i_*(*t*_0_), *δ_i_* (*t*+ *t*_0_)), becomes *ρ*(*t*) = exp{−|*t*|/*τ*}, where *τ* describes the rate of decay for the correlation function for time between observations of |*t*|. This structure assumes that observations farther apart in time are less correlated. The vector ε_i_ can be expressed as *ε_i_* ~ *MVN*(0, **Σ**), a multivariate normal density with mean **0** and variance-covariance matrix **Σ**. Another similar choice for the covariance is to specify the Gaussian correlation function. Other options are available to implement in standard software for mixed models [11,12].

### Knot and spline selection

Depending upon the nature of the data, there are a variety of spline basis expansions that could be used to represent the mean response function, f(t), in Equation (1). The basis function expansion for f(t) can be generally defined as:

(3) $$f\left(t\right)=\sum_{r=1}^{R}\beta_{r}b_{r}\left(t\right)$$

Parameters β_1_,… β_R_ are the coefficients of the expansion with basic functions b_1_(t),… b_R_(t). These spline bases are piecewise polynomials constructed by segmenting intervals over the range of t into subintervals. Basis functions over these subintervals are joined by knots K_1_,…K_K_, which are points along the time interval of interest. The degree of polynomial may depend upon available sample size (both number of subjects and number of time points the sequence is sampled) and inferential goals. If we are interested in taking derivatives of f(t), then polynomial degree must be considered. On the other hand, specifying a dense grid of knots along the time interval but using lower degree polynomials in Equation (3) may be sufficient to estimate curvature. Note that the number of basic functions (*R*) will depend on the total number of knots (K), the type of basis function employed, and the highest degree of the polynomial used in the expansion.

There are several options available for basic functions. Most notable are B-splines, natural cubic splines and penalized splines. Linear, quadratic and cubic B-splines have remained a popular choice due to their numerical stability, particularly if fitting f(t) using ordinary least squares (OLS). For example, if we specify a cubic B-spline basis joined by *K* knots, then Equation (3) will have R=K+3 basis functions. Another common choice that can be viewed as a special modification of the cubic B-spline is the natural cubic spline. Natural cubic splines have the same properties as cubic B-splines but impose the additional constraint that the basic functions are linear to the left and right of the boundary knots, K_1_ and K_K_. This constraint reduces potential for over-fitting and computational errors due to singularity issues in applications where data are sparse at time points outside of boundary knots [8].

In semiparametric mixed models, it is common to specify basis functions originating from scatterplot smoothers posed, which were posed as early as 1985 by Parker and Rice [13] and are known in more recent literature as penalized splines [7]. A common spline basis expansion for semiparametric mixed modeling is the p^th^-degree truncated power basis [14], which corresponds to penalized splines of the form:

(4) $$f\left(t\right)=\beta_{0}+\beta_{1}t+\ldots +\beta_{p}t^{p}+\sum_{k=1}^{K}b_{k}{\left(t-\kappa_{k}\right)}_{+}^{p}$$

The polynomial terms β_0_, β_1_… β_P_ are the traditional coefficients for the respective intercept, slope and higher-order terms. The summand expression is comprised of the basic functions b_1_,…b_K_, joined by knots K_1_,…K_K_, and evaluated at time t. The expression (t-k)_+_ is the positive part of function t-k because “+” sets it to zero when t<k. The truncated power function ${\left(t-\kappa \right)}_{+}^{p}$ leads to smoother spline functions with higher values of p. It can be shown that penalized splines defined in Equation (4) are mathematically equivalent to a B-spline basis expansion of the same polynomial degree.

Numerous algorithms are available to aid in selecting the number and location of knots, ranging from automated approaches [7] to adaptive algorithms [15]. While adaptive algorithms are more attractive due to computational advancements, there are numerical disadvantages [16]. Sparse data at specific time intervals may create singularities, which can cause computational errors during fitting [17]. Previous work describes the implementation of penalized-spline smoothing in mixed models, thereby providing an automated approach to obtaining the degree of smoothing to avoid under- or over-fitting data [18, 19].

### Estimation

By representing f(t) using penalized splines, estimation can be conducted via penalized least squares, where a penalty term induces smoothness. Linear mixed models (LMMs) provide a framework for estimating both parametric fixed effects and random effects [20] and have been a mainstay in longitudinal data analysis [21]. If we restrict our attention to modeling the time effects in Equation (4), the LMM including *p*-splines can be expressed in matrix form as

(5) $$Y=X\beta \underset{f(t)}{\underset{︸}{Z_{b}b}}+\underset{\varepsilon^{*}}{\underset{︸}{Z_{u}u}}+\varepsilon$$

The design matrix X and corresponding fixed-effects vector β represent the polynomial terms; Z_b_ and b correspond to the matrix for the *p*th-degree spline basis functions and coefficients in the summand in Equation (4). Typically, the spline basis functions are assumed to follow a Gaussian distribution such that $b_{k}\sim N\left(0,\sigma_{b}^{2}\right)$, and may be thought of as smooth deviations from the global polynomial terms represented as fixed effects. The remaining terms of Equation (5) are used to specify the more conventional random effects in the LMM and covariance structure. Random effects terms for subject-specific variation may be relaxed to accommodate subjects with outlying observations. Alternatives to the Gaussian distribution assumption for random effects include the use of multivariate t-distributions [22] and truncated Dirichlet process [23].

Fitting semiparametric mixed models in the LMM framework is a consequence of the Bayesian model posed by Wahba [24]. Conditional on restricted maximum likelihood (REML) estimates of the smoothing parameter terms in Equation (4) and variance of b_k_, the spline estimates are regarded as empirical best linear unbiased predictions (EBLUPs) [25]. Furthermore, REML has been shown to be equivalent to generalized maximum likelihood, which is an optimal criterion for smoothing parameter selection in the presence of correlated data [26].

Despite the connection between the LMM and spline smoothing [27], REML estimation has an unexplained sensitivity knot specification [28], particularly in semiparametric additive models [7]. This could be problematic if we wish to add continuous smooth functions of other predictors to Equation (1). Parameter estimates obtained from a series of semiparametric mixed models with different structures for the fixed effects cannot be appropriately compared via the conventional likelihood ratio test (LRT) using the Chi-square test statistics across models if REML estimation is employed; however, REML estimation may be used by replacing the Chi-square test statistic with the corresponding *F* test statistic [29].

Although REML is commonly used and particularly well suited for variance component estimation [20], maximum likelihood (ML) has also been used for parameter estimation [29]. Semiparametric mixed models can also be estimated using nonparametric Bayesian analysis, where all parameters are treated as random. Implementation of this approach has already been provided for instances where the number and location of knots are specified a priori [30]. Selecting the appropriate method often depends on how the hypotheses are formulated in terms of the research question and application of interest.

### Model selection, diagnostics and goodness-of-fit

The model selection problem in this setting is typically two-fold, focusing on 1) whether the inclusion of spline effects improves model fit, compared to a fully parametric model; 2) optimal selection of candidate predictors. The first problem, accurate identification of nonlinear features, corresponds to testing linearity of f(t). One approach is to use the restricted LRT test to assess the significance of the variance component $\sigma_{b}^{2}$ in Equation (5). This restricted LRT has been developed for testing the inclusion of linear penalized spline effects, but issues arise if we wish to test polynomials against a spline alternative [31]. Alternatively, we could use an approximate F-test for semiparametric models [32], in which residual degrees of freedom for the null and alternative model fits are used in place of the number of parameters in each model. A third option, which has been shown to yield different conclusions [7], is to use simulation for *P-*values obtained via the LRT or F-test.

The second challenge arising from model selection is the more familiar covariate selection issue, which arises when there are several candidate predictors in Equation (1). The nonparametric component in the model adds further complexity to the usual selection approaches. Some work has been done to extend stepwise algorithms to semiparametric models [33]. Selection may be further complicated if the model includes more than one predictor being fitted as a continuous smooth function, since conclusions may be sensitive to degrees of freedom chosen across multiple predictors. More recent work on simultaneous model selection and estimation in semiparametric mixed models provides a unified approach that can be implemented using the LMM routines in existing software [34].

Information criteria with adjustment for penalized splines are available for semiparametric mixed models. An adjusted Akaike’s information criterion (AIC) for model selection has been proposed [35]. A term to estimate the effective number of parameters is added to the marginal AIC typically reported using LMM routines. This term penalizes models with splines, thereby providing appropriate inflation for the additional parameters. A modified version of the Bayesian Information Criterion (BIC) is now available for semiparametric additive models [36]. Influence diagnostics available in the traditional LMM have been extended to the semiparametric stochastic mixed model. Fung and colleagues [37] developed influence diagnostics based on case deletion to examine influence of an individual observation on parametric fixed effects and the nonparametric function in Equation (1). They provided a generalized Cook’s distance measure of the influence on θ and a DFIT measure to examine partial influence on f(t). These influence measures are directly computable if using the estimation technique by Zhang and colleagues [10].

## Model Inference

### Estimating rapid changes in the smooth function

Once an appropriate semiparametric mixed model has been selected, it is often of interest to determine how the smooth function, f(.), is changing over time. We can estimate changes in the smooth function by taking the derivative with respect to time. Taking the first-order derivative of the penalized splines in Equation (4) yields:

(6) $$f^{\prime }\left(t\right)=\beta_{1}+\beta_{2}t+\cdots +\beta_{p}t^{p-1}+p\sum_{k=1}^{K}b_{k}{\left(t-\kappa_{k}\right)}_{+}^{p-1}$$

An estimate of the derivative at time t, denoted *f̂*′(*t*), can be obtained by substituting parameter estimates *β̂*_1_,…, *β̂_p_* and *b̂*_1_, …, *b̂_K_* in Equation (6). This “plug-in” approach is analogous for other penalized spline representations.

Generally, higher-degree polynomial terms are necessary to obtain smooth estimates of *f*′. Choosing such higher-degree basis functions may not lead to optimal choices for smoothing f. Performing this smoothing in the LMM depends only on ${\hat{\sigma }}_{b}^{2}$ and is independent of derivative order. Testing for existence of a particular feature often requires estimating *f*′(*t*) over a specific interval of time. Chaudhuri and Marron developed systematic approaches to feature significance selection [38], primarily focused on estimating inflection points in smooth functions. A compound estimator that is consistent for f(t) and its derivatives have been proposed in more recent work [39].

### Testing group-by-curve interactions

It is desirable to have an approach for comparing group-specific smooth functions over time. Early developments focused on smoothing spline analysis of variance models for longitudinal data [40, 41] and mixed effects formulations with nonparametric model fitting [42]. We can accommodate group-specific smoothing functions by replacing f(t) in Equation (4) with *f*_*z*_*i*__(*t*), where *z_i_* ∈{1,… *L*} indicates group membership for the i^th^ subject. Group-specific smoothing is also possible by representing group effects as levels of a categorical covariate in the LMM in Equation (5) and allowing separate spline coefficients and smoothing parameters by each level [43]. Using this approach, group specific differences can be tested by having the null hypothesis correspond to *H*_0_ : *f_l_*(*t*) = *f_l_*_′_(*t*), versus the alternative hypothesis corresponding to *H*_1_ : *f_l_*(*t*) ≠ *f_l_*_′_(*t*), for *l* ≠ *l*′. A series of alternative hypotheses for testing H_1_ were presented in more recent work [29]. This corresponds to a class of semiparametric mixed models to test group effects, which we subsequently illustrate with our case study.

### Constructing confidence bands

We do not need to independently test differences between groups across several time points within the 24-hour interval. Instead, we can augment existing results [7,29] to construct simultaneous confidence bands of differences between group-specific smooth functions *f_l_*(*t*) and *f_l_*_′_(*t*) where (*l* ≠ *l*′) under the aforementioned semiparametric mixed model in Equation (5) and its covariance formulation

(7) $$\mathrm{Cov}\left[\begin{array}{c} b\\ \varepsilon^{*} \end{array}\right]=\left[\begin{array}{cc} G & 0\\ 0 & R \end{array}\right]:\left[\begin{array}{c} \hat{\beta }-\beta \\ \hat{b}-b \end{array}\right]\sim N\left\{0,{\left(C^{T}{\hat{R}}^{-1}C+\hat{B}\right)}^{-1}\right\}$$

Where $C=\left[\begin{array}{cc} X & Z_{b} \end{array}\right]$ and $B=\left[\begin{array}{cc} 0 & 0\\ 0 & G^{-1} \end{array}\right]$. This normal distribution is conditional on variance component estimators. Let **g** = (g_1_, …, g*_T_*) be a grid of equally spaced time points. We evaluate the estimated difference between the two functions as

(8) $${\hat{f}}_{d}-f_{d}={\hat{f}}_{l}-{\hat{f}}_{l^{\prime }}-\left(f_{l}-f_{l^{\prime }}\right)=C_{g}\;\left[\begin{array}{c} \hat{\beta }-\beta \\ \hat{b}-b \end{array}\right]=\left[\begin{array}{cc} \mathrm{LX} & LZ_{b} \end{array}\right]\;\left[\begin{array}{c} \hat{\beta }-\beta \\ \hat{b}-b \end{array}\right]$$

where $L={\left(\begin{array}{cccccc} 1 & \; & \; & -1 & \; & \;\\ \; & \ddots & \; & \; & \ddots & \;\\ \; & \; & 1 & \; & \; & -1 \end{array}\right)}_{T\times 2T}$ is a contrast matrix; X and Z_b_ are the design matrices evaluated over [**g**]; $C_{g}=\left[\begin{array}{cc} \mathrm{LX} & LZ_{b} \end{array}\right]$.

In order to obtain the *SD*[***f̂****_d_* − ***f****_d_*] for the confidence bands, it is necessary to compute *Cov*[***f̂****_d_* − ***f****_d_*] as

$$\mathrm{Cov}\left[{\hat{f}}_{d}-f_{d}\right]=C_{g}\;\mathrm{Cov}\left(\left[\begin{array}{c} \hat{\beta }-\beta \\ \hat{b}-b \end{array}\right]\right)\;{C_{g}}^{\prime }=C_{g}{\left(C^{T}R^{-1}C+B\right)}^{-1}\;{C_{g}}^{\prime },$$

Then we have $SD\left[{\hat{f}}_{d}-f_{d}\right]=\sqrt{\text{the}\;\text{diagonal}\;\text{elements}\;\text{of}\;\mathrm{Cov}\left[{\hat{f}}_{d}-f_{d}\right]}$. The 95% point wise confidence bands for f_d_ are [***f̂****_d_* ± *Z*_0.95_*SD*{***f̂****_d_* − ***f̂****_d_*}]_1≤_*_l_*_≤_*_T_*, where Z_0.95_ = 1.96. The point wise confidence bands are based on an asymptotic approximation and may not be always accurate in practice. To improve the accuracy, we use a straightforward, simulation-based method and obtain the 95% simultaneous confidence bands for ***fd*** as:

$${\left[{\hat{f}}_{d}\pm h_{0.95}SD\left\{{\hat{f}}_{d}-f_{d}\right\}\right]}_{1\leq l\leq T},$$

Where h_0.95_ is the 1-α quantile with α = 0.05 of the random variable:

(9) $$\sup\left|\frac{{\hat{f}}_{d}\left(t\right)-f_{d}\left(t\right)}{\hat{SD}\left\{{\hat{f}}_{d}\left(t\right)-f_{d}\left(t\right)\right\}}\right|\approx \max_{1\leq l\leq T}\left|\frac{{\left(C_{g}\left[\begin{array}{c} \hat{\beta }-\beta \\ \hat{b}-b \end{array}\right]\right)}_{l}}{\hat{SD}\left\{{\hat{f}}_{d}\left(g_{l}\right)-f_{d}\left(g_{l}\right)\right\}}\right|.$$

The quantile h_0.95_ can be approximated using simulations. As an example, if simulations from Equation (7) then computations of Equation (9) are repeated 10,000 times, the value of the ranked 9,500^th^ quantity is used as h_0.95_. This approach may also be used to construct simultaneous confidence bands for previously described derivatives of each smooth function by replacing ***f̂****_d_* and f_d_ in Equation (8) with ***f̂***′ and ***f***′ defined in Equation (6). Confidence bands based on bootstrap computation, called bootstrap confidence bands, also use the simulation-based method to reduce the coverage error. They are applicable to small data sets, large data sets and complex models and are developed for both parametric and nonparametric situations. They can also be applied to our case. However, this method has greater computational expense than the point wise and simultaneous confidence bands. More details of bootstrap confidence band methods are available in previous seminal work [44] with additional discussion [45].

## Semiparametric Mixed Model Applications in the Literature

Maringwa and colleagues [29] developed semiparametric mixed models to evaluate differences between group-specific curves in a longitudinal study of cardiovascular functioning. The models were applied to QT prolongation data for the purpose of assessing differences between intervention and control groups. Briefly, the QT interval is used to measure electrical activity of the heart ventricle. Having a prolonged QT interval is associated with increased risk of ventricular arrhythmias. Using the LMM framework, they were able to specify semiparametric mixed models that correspond to various scenarios with differences between group-specific curves. Their formulation utilized the linear truncated power basis, which corresponds to p=1 in Equation (4). The series of stepwise models, proposed as alternative hypotheses to no group effect, begin with intercept-only differences (*H*_1_: *β*_0_*_A_* ≠ *β*_0_*_B_*), then add slope-specific differences (*H*_1_ : *β*_1_*_A_* ≠ *β*_1_*_B_*), spline-coefficient differences ( $H_{1}:b_{k}^{A}\neq b_{k}^{B}$), and finally differences in the degree of smoothing inherent in the variance components $\left(H_{1}:\sigma_{bA}^{2}\neq \sigma_{bB}^{2}\right)$. The authors note that a LRT with the appropriate Chi-square distribution may be used to compare each developed model to the common model [20], because the inference focuses on the fixed effects only. Once a model was selected for mean response functions of QT prolongation over time, the authors employed simultaneous confidence bands to assess differences between groups over the continuum of time. This stepwise-modeling approach has been used to assess differences in glycemic control monitored over time between clinically distinct groups of pregnant mothers with type 1 diabetes [46].

In another study, Maringwa and colleagues developed and applied a semiparametric mixed model to QT prolongation data arising from a crossover design, in order to compare condition-specific mean profiles over the continuum of time [47]. Correlations between and within periods were assumed to be separable [48]. In this context, between-period measurements were accounted for via subject-specific random intercepts while different correlation structures were considered for within-period measurements. In another study under review, we examined these assumptions and other potential covariance models in nested repeated measures experiments.

More recent work utilized semiparametric mixed models and first-order derivatives to characterize the timing and degree of rapid lung function decline in individuals with cystic fibrosis lung disease [49]. In this model of longitudinal disease progression, additional considerations were made to depict the serial correlation inherent in measurements of lung function and assess the rate of change in lung function decline (known clinically as the degree and timing of rapid decline). The authors used three separate, presumably independent effects to model stochastic variation about the mean response function.

### Case study

It is well known in the clinical community that 24-hour blood pressure monitoring allows for examination of several blood pressure measures. The rate of change in blood pressure from sleep to wakefulness, commonly referred to as “morning surge,” is a marker of cardiovascular function. Another feature of interest is nocturnal dipping of blood pressure. A significant drop in blood pressure during the period of sleep indicates the presence of this feature. It is of great interest in hypertension and pulmonary research to understand how these features may differ in children, who have severe obstructive sleep apnea (OSA), compared to their otherwise healthy counterparts. OSA is diagnosed using the obstructive index (OI) calculated from overnight polysomnography data. The OI for each individual is an average that typically represents the combined number of apneas and hypopneas, also known as obstructive events, which occur per hour of sleep. For childhood evaluation, 1≤OI<5 represents the milder range of sleep apnea, while OI≥5 corresponds to more severe sleep apnea.

As an illustrative example, we analyze data previously described by Amin and colleagues (1) and also by Crisalli and colleagues [50]. Briefly, each subject enrolled in the study wore an ambulatory blood pressure monitoring cuff for a period of no less than 36 hours. Actigraphy data were also collected during this time period and used to determine the time of sleep onset (denoted in our case study as t=0). The primary aim was to determine whether group-specific differences existed in the mean ambulatory blood pressure responses of children with OSA and otherwise healthy controls, monitored over a 24-hour period. Clinical and demographic data collected include age, height, weight, gender and race. Although not presented here for our illustrative example, data were also acquired to measure vascular function and cytokine levels. Study data are not publicly available. Institutional IRB approval from Cincinnati Children’s Hospital was obtained for the original study protocol; subsequent approval via IRB exempt review was granted for the analyses presented here.

**Figure 1:**
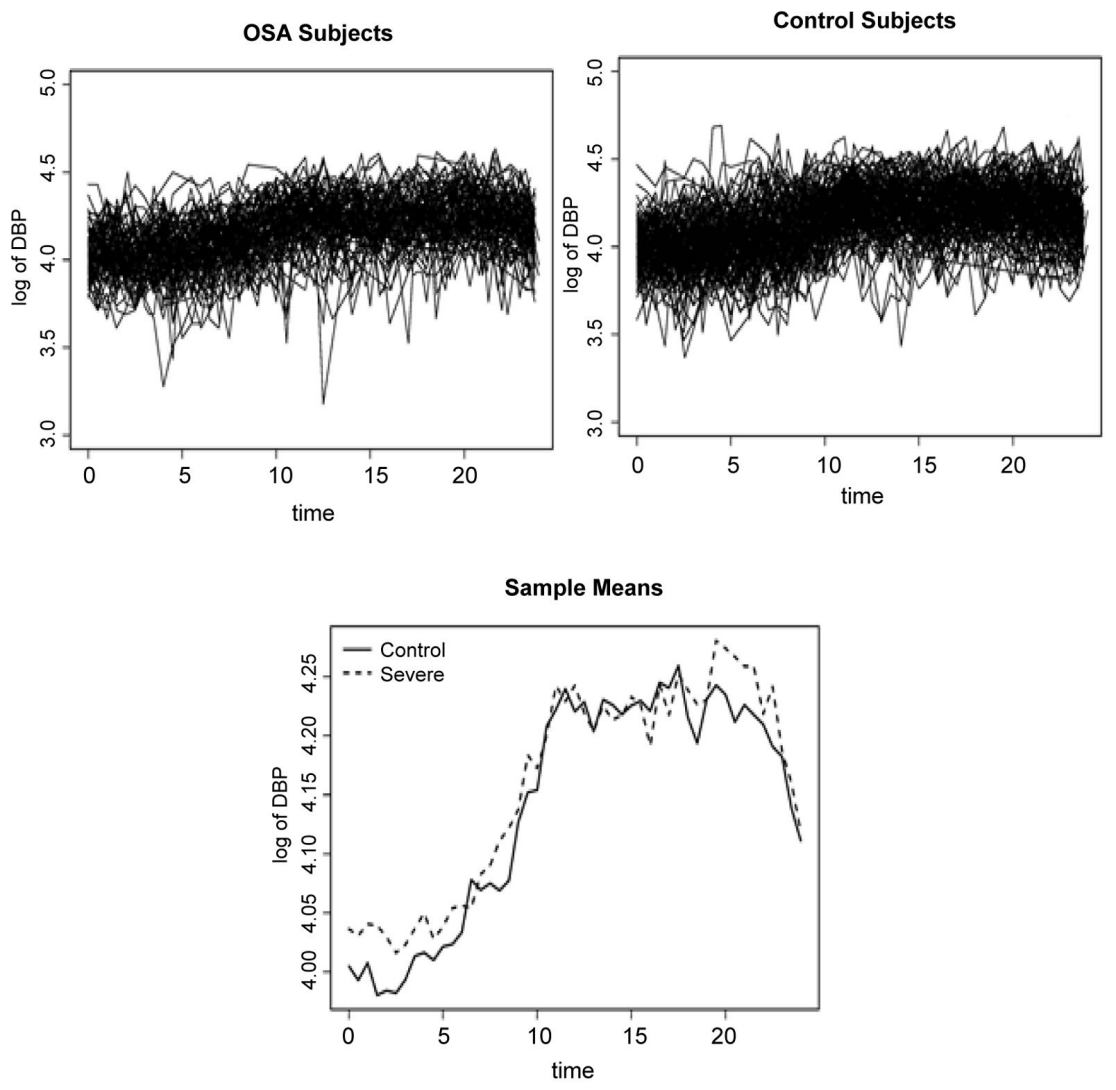
Observed diastolic blood pressure (DBP), on the log scale, beginning with sleep onset and ending after 24 hours. Clockwise from upper left: Individual profiles for subjects separately from 87 subjects in the severe obstructive sleep apnea (OSA) group (upper left) and 135 Control subjects (upper right) measured in the study; sample means for Control (solid line) and severe OSA (dashed line) groups at each half hour.

Diastolic blood pressure (DBP), a response of interest in the clinical study, was collected at half-hourly intervals on each subject. Each subject’s DBP recording was aligned with respect to time of sleep onset. For our case study, we utilize data obtained during the 24-hour period from sleep onset. We focus on the following two experimental groups from this study: subjects with an OI above 5 events per hour (classified as having severe OSA) and those subjects with less than 1 event per hour (classified as Controls). The individual DBP profiles are shown in Figure 1. Although nocturnal dipping may be evident in a few of the subjects, these data exhibit both substantial within- and between-subject variability.

Inferential goals of our case study include 1) evaluating group-specific differences in mean DBP response functions over the twenty-four hour interval; 2) characterizing rate of change in the response functions. Assuming no group effect, we can formulate a semiparametric mixed model for monitored DBP as:

(10) $${\text{DBP}}_{ij}=f\left(t_{ij}\right)+\sum_{m=1}^{M}\theta_{m}x_{\mathrm{ijm}}+u_{i}+\varepsilon_{i}\left(t_{ij}\right)\;f\left(t_{ij}\right)=\beta_{0}+\beta_{1}t_{ij}+\beta_{2}{t_{ij}}^{2}+\sum_{k=1}^{K}b_{k}{\left(t_{ij}-\kappa_{k}\right)}_{+}^{2}$$

First, the expression in the summand represents the covariate effects and parameters θ_1_,…θ_M_; these include age (in years), z-score for the body mass index (denoted BMIZ), gender and race (dichotomized as white/not white); u_i_ is a random intercept for the i^th^ subject, and characterizes how the overall mean DBP response function varies from subject to subject, following a Gaussian distribution with mean 0 and variance $\sigma_{u}^{2}$, denoted as $u_{i}\sim N\left(0,\sigma_{u}^{2}\right)$. The term *ε_i_*(*t_ij_*) represents the within-subject variation, which is comprised of exponential correlation function and measurement error described in the general model framework. The response function evaluated at time t_ij_, expressed as f(t_ij_), is defined as quadratic penalized splines and may be thought of as the mean function for covariate values equal to zero. The spline formulation is equivalent to p=2 in Equation (4).

The semiparametric mixed model in Equation (10) corresponds to the null hypothesis defined as *H*_0_: *f_OSA_*(*t*) = *f_CTR_*(*t*)in the stepwise approach. We evaluated the class of semiparametric mixed models in Table 1. Model (1.1) corresponds to no difference between OSA and Control groups. Each successive model represents an alternative hypothesis of the form *H*_1_ : *f_OSA_*(*t*) ≠ *f_CTR_*(*t*), in order to assess group differences in mean response functions over time between the OSA and Control groups. Model (1.2) indicates the group differences are parallel over time. Model (1.3) provides separate intercept, linear and quadratic polynomials for the difference between groups, which suggests the group difference follows a global quadratic trend over time. Model (1.4) allows for subtler group differences by having different sets of spline coefficients for OSA and Control groups. Finally, Model (1.5) adds separate smoothing and distinct smoothing parameters in the form of separate variances for the spline coefficients of OSA and Control groups ( $\sigma_{b^{\mathrm{OSA}}}^{2}$ and $\sigma_{b^{\mathrm{CTR}}}^{2}$).

**Figure 2:**
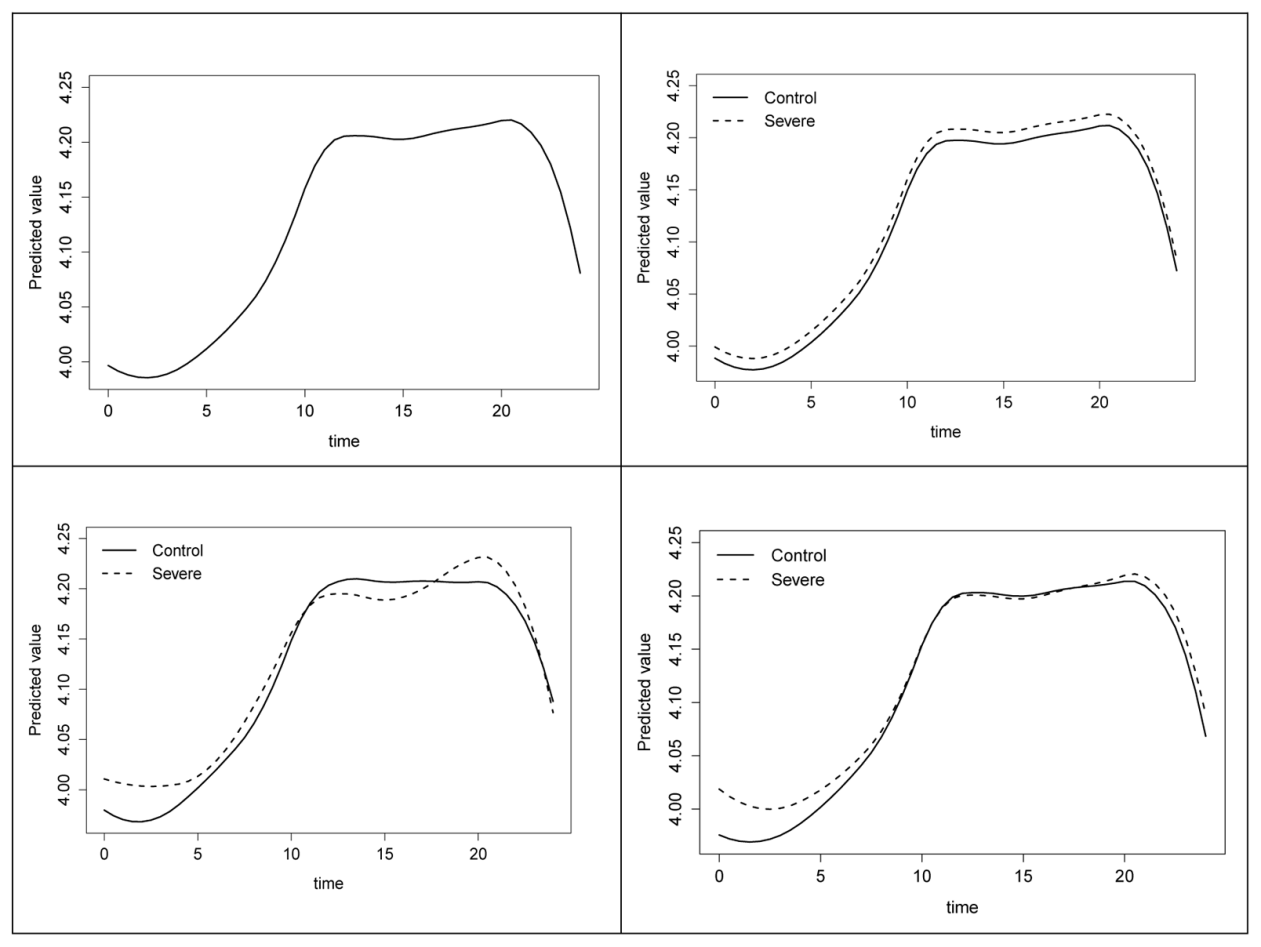
Hypothesized differences between groups for semiparametric mixed models applied to the diastolic blood pressure (DBP) data. Beginning clockwise from the upper left panel, smooth function obtained from model assuming a common profile (Model 1.1); smooth functions with parallel group differences over time (Model 1.2); distinct quadratic trends (Model 1.3); different smoothing on functions via distinct spline coefficient vectors for the groups (Model 1.4).

SAS implementation for each model is available as Supplemental Information. This implementation is based on previous approaches [18, 19]. Given our specific interest in rate of change for the smooth functions, we chose to use quadratic penalized splines to model the DBP mean response. We selected the number and location of knots using the quantile-based approach described by Ngo and Wand [18]. Knots used across the 24-hour interval included k=(3.95, 7.20, 9.77, 11.98, 13.98, 16.03, 18.10, 20.01, 22.05). The fitted values from Models (1.1)-(1.4) are presented in Figure 2 and show how the different specifications can produce a variety of trends for the mean DBP response function over time. All four models suggest the presence of nocturnal dipping, which indicates that DBP drops during sleep; however, Models (1.2)–(1.4) have different levels of emphasis on how distinct the nocturnal dipping patterns are between Control and OSA groups. Each of these three models suggests there may be periods across the 24-hour interval in which the mean DBP response function for the OSA group is slightly elevated, compared to the Control group.

Fit statistics are presented in Table 2 for Models (1.1–1.5) and each included subject-specific random intercepts. Adjusted AIC shows that Model (1.3) had the smallest value, implying that, out of the models evaluated, group differences over time are best characterized by having separate intercept, slope and polynomial terms for OSA and Control groups. As shown for adjusted AIC, the penalty term E_p_ increased as complexity of the spline formulation increased, but dropped off in Model (1.5), which specified different spline variance components for OSA and Control groups. This drop in E_p_ was somewhat unexpected and suggested results were questionable under this model. Fit statistics were similar for Models (1.1)–(1.4); however, Model (1.5) had fit statistics that were higher than the other models. Estimates of $\sigma_{b^{\mathrm{OSA}}}^{2}$ and $\sigma_{b^{\mathrm{CTR}}}^{2}$ in Model (1.5) were close to the zero boundary. Due to lack of model fit, graphical results are not displayed for Model (1.5). Although our presented results are based on ML estimates, the results with REML estimates yielded similar conclusions across all models.

We proceeded to covariance model selection using Model (1.3) as the basis for all effects excluding the error term ε^*^ in Equation (5). For our illustration, we examined three different covariance models for the DBP data (Table 3). Of those three covariance models examined, the exponential covariance model was found to have the smallest adjusted AIC. We found that stepwise model selection results in Table 1 did not change if the exponential correlation function was specified. Model (1.3) with exponential covariance provided the best fit, with respect to information criteria presented in Tables 1–2, of all semiparametric and covariance affects structures considered.

**Figure 3:**
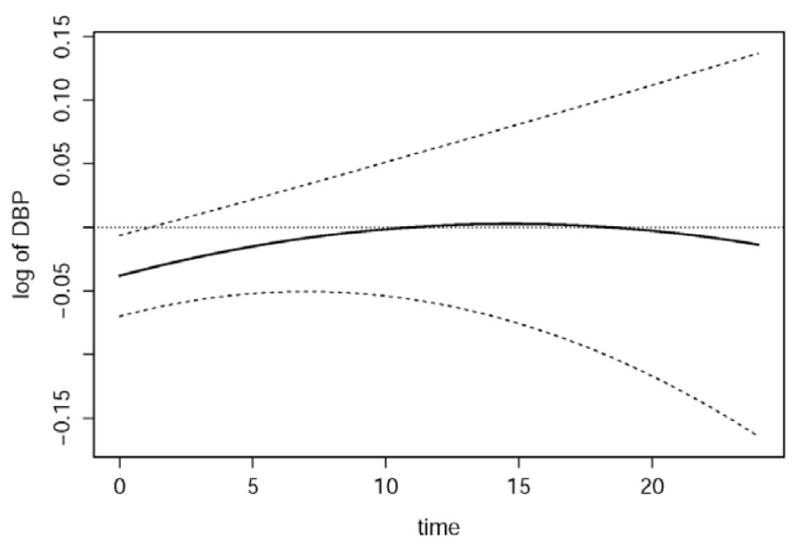
95% simultaneous confidence bands for temporal differences between OSA and Control mean response functions obtained from selected semiparametric mixed model. Differences are constructed as *f̂_CRT_*(*t*) − *f̂_OSA_*(*t*).

We investigated group-specific differences between the mean response functions using 95% simultaneous confidence bands for the selected model. We defined a grid g of time points (0, 24) by increments of 0.5 hours such that there are T=49 equally spaced time points (g_1_, …, g_49_)′. The resulting simultaneous confidence bands for differences between group-specific functions are in Figure 3. The point wise bands were similar to displayed simultaneous bands; as expected, however, these bands have decreased width compared to simultaneous bands. Although the bands suggest statistical significance for only a brief duration of the 24-hour interval, they do suggest that mean DBP response for the OSA group may be slightly elevated, compared to the Control group, during sleep. The confidence bands become markedly wider as the 24-hour interval proceeds, demonstrating the increased variability. This increase may be due to a smaller number of blood pressure recordings being observed at the later period of the interval or a potential artifact induced by the spline smoothing.

**Figure 4:**
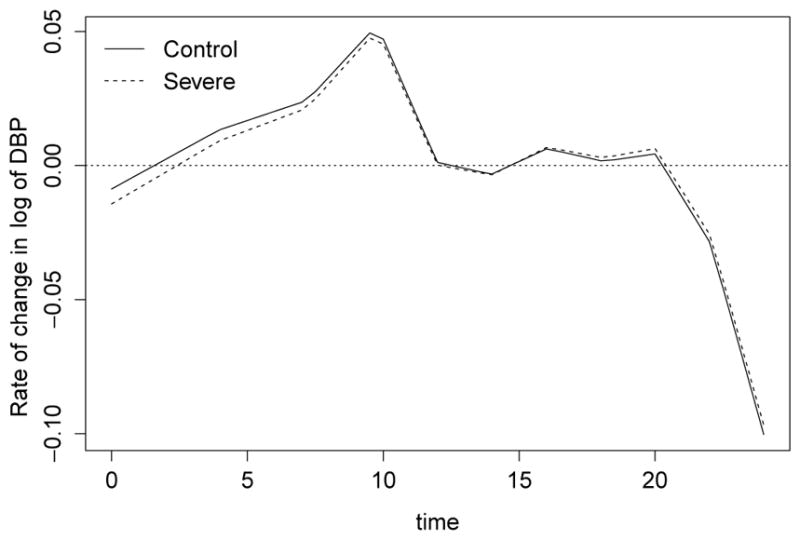
Rate of change for selected semiparametric mixed model of OSA and Control mean response functions for diastolic blood pressure.

The derivatives $f_{\mathrm{OSA}}^{\prime }\left(t\right)$ and $f_{\mathrm{CTR}}^{\prime }\left(t\right)$ taken from the functional forms in Model (1.3) can be used to examine the morning surge separately for the OSA and Control groups. By looking at time since sleep onset, which corresponds to 0 on the t-axis in Figure 4, the rate of change in the mean DBP response is slightly higher for the Control group; however, the derivative curves begin to overlap around t=9 hours after sleep onset. Presumably, 7–9 hours after sleep onset is the interval of interest to assess the morning surge, as this is the time frame when subjects begin to wake. Results suggest the rate of change is slightly elevated in the Control group, as compared to the OSA group. From a biomedical perspective, this finding may indicate that children without OSA have a somewhat dampened response to wakefulness. Despite the clinical implications, these results are not statistically significant. The 95% simultaneous confidence bands are not shown but could be derived similarly to those we obtained for *f_CTR_*(*t*) − *f_OSA_*(*t*) in Figure 3.

### Small simulation study

In addition to our case study, previous studies utilizing the stepwise modeling approach also indicate that group effects as specified in Model (1.3) have the best fit of all considered models, as measured by adjusted AIC (29, 46). We further explored the sensitivity of this selection strategy by conducting a small simulation study and evaluating models on the basis of the adjusted AIC. We generated data by setting fixed effects values equal to estimates obtained from fitting Model (1.5) to our case study data. The error variance was also set equal to the model estimate. We generated spline coefficients separately by group according to variance values from the Uniform distribution (min: 0, max: 1).

We performed 1,000 simulations under these settings and found that Model (1.3) was always selected as the optimal model based on fit statistics. We slightly modified these results to examine behavior of the model selection process when variance components for the spline coefficients, which enter into Model (1.5), had true values near the zero boundaries. As it turns out, incorporating these differences still led to Model (1.3) being most favorable of the models considered. Our preliminary findings suggest that additional work may be needed to develop a more sensitive indicator of model fit when a class of semiparametric mixed models is evaluated and also when differences in degree of smoothing are truly evident.

## Discussion and Conclusion

In this paper, we have reviewed work demonstrating that semiparametric mixed modeling within the LMM framework is a flexible method for modeling data arising from medical monitoring. Furthermore, this approach can accomplish important inferential goals common to these settings. We have discussed several inferential tools related to spline structure and covariance model selection. We illustrated the approaches and investigated available model inference techniques using a case study with medical monitoring data and included implementation in mixed model software.

There are alternative approaches to the function representations illustrated in our case study. Blood pressure patterns naturally follow a circadian rhythm with twenty-four hour periodicity [51]. It is possible to express the model in terms of clock time and use trigonometric basis functions to represent f(t). For example, Wang and colleagues model actigraphy data, which also exhibits twenty-four hour periodicity, by using a Fourier expansion [52]. Our primary interest was to model blood pressure patterns in relation to time since sleep onset, which may correspond to an aperiodic f(t) since some individuals may have sleep and wake times that differ from day to day. Our case study also has limitations related to spline smoothing and development of confidence bands. In parametric regression, observed errors may be replaced by residuals from the fitted model. Spline smoothing creates an additional challenge in semi- or nonparametric regression settings through the inherent bias from using truncated basis functions in practice. The confidence bands obtained using the approach from the case study does not account for this truncation bias; therefore, these bands are symmetric about the fitted function and may be questionable. Approaches are available to correct for this bias, ranging from bootstrapping methods [53] to incorporating corrections for f(t) and its derivatives [54].

Even though we have tried to review the most relevant aspects of semiparametric mixed models applied to medical monitoring data, there are inevitably a number of topics and extensions that have not been touched upon. The longitudinal processes arising from medical monitoring can involve failure-time data, such as disease onset combined with biomarker data [55]. We focused on settings in which a single longitudinal process is observed, but the models presented can be expanded to include nonparametric functions for other covariates. It is also possible to extend the semiparametric mixed models presented here to allow subject-specific smooth functions of the form f_i_(t) [56]. These individual functions may be represented as functional mixed effects [25]. If the individual functions can be related to an underlying shape function, self-modeling regression may provide additional information on subject specific deviations from the mean response function [57]. This approach has been extended to include a penalized spline formulation and covariance models for longitudinal data [58]. In an application to growth curve fitting, the self-modeling coefficients were treated as subject-specific random effects [59].

While the ease of implementation makes fitting semiparametric mixed models appealing for medical monitoring and other continuous longitudinal data sources, there still exist several non-trivial issues. For example, although classes of semiparametric mixed models have been developed and an optimal model can be selected using established information criteria, the traditional LRT yielded somewhat disappointing results in our small simulation study. It is possible that the underlying data-generation mechanism is the issue and requires further investigation. Another challenge in semiparametric mixed model development is selecting the location and number of knots. Early work found that specifying the number of knots and their locations a priori works well for most data applications [18]. Adaptive methods have not been pursued to a large extent in semiparametric mixed modeling due to their computational intensity, but knot selection algorithms such as Bayesian adaptive regression splines (BARS) have been used in biological studies with continuous longitudinal data [60] and extended for other functional data applications, such as clustering self-modeling data [61].

Semiparametric mixed models have clinical utility, as shown in the studies referenced in our overview, but distinctions need to be made between prospective medical monitoring and other settings. For example, if the models are being developed for prognostic purposes but are based on a clinical patient registry, then statistical causality should be incorporated. This could be achieved by including latent variables to characterize the causal structure of the model [62]. Another aspect to advancing the use of semiparametric mixed models in clinical settings would be to improve their real-time assessment. Joint modeling has been proposed as an approach to improve real-time prediction [63]. Recent work involves the use of Gaussian process regression to achieve improvements [64], which eliminates the need for knot selection and spline development. Real-time semiparametric regression was recently proposed by Luts and colleagues [65] and includes feasible algorithms for prognostic assessments. There are several expansions that could be made to this methodology, in order to account for changing trends in clinical care while providing flexible models that offer increased clinical utility.

## Figures and Tables

**Table 1:** Class of semiparametric mixed models to evaluate group-specific differences in twenty-four hour diastolic blood pressure.

Effects Description	Mean Response Structure*
(1.1) No group difference	$\beta_{0}+\beta_{1}t_{ij}+\beta_{2}t_{ij}^{2}+\sum_{k=1}^{K}b_{k}{\left(t_{ij}-\kappa_{k}\right)}_{+}^{2}$
(1.2) Difference between groups constant across 24-hour sequence	$\beta_{0}+\beta_{02}{\text{OSA}}_{i}+\beta_{1}t_{ij}+\beta_{2}t_{ij}^{2}+\sum_{k=1}^{K}b_{k}{\left(t_{ij}-\kappa_{k}\right)}_{+}^{2}$
(1.3) Group-specific mean response functions have different quadratic trends	$\beta_{0}+\beta_{02}{\text{OSA}}_{i}+\beta_{1}t_{ij}+\beta_{12}{\text{OSA}}_{i}t_{ij}+\beta_{2}t_{ij}^{2}+\beta_{22}{\text{OSA}}_{i}t_{ij}^{2}+\sum_{k=1}^{K}b_{k}{\left(t_{ij}-\kappa_{k}\right)}_{+}^{2}$
(1.4) Group-specific mean response functions smoothed differently using distinct vectors of coefficients for OSA and Control profiles	$\beta_{0}+\beta_{02}{\text{OSA}}_{i}+\beta_{1}t_{ij}+\beta_{12}{\text{OSA}}_{i}t_{ij}+\beta_{2}t_{ij}^{2}+\beta_{22}{\text{OSA}}_{i}t_{ij}^{2}+\sum_{k=1}^{K}b_{k}^{{\mathrm{OSA}}_{i}}{\left(t_{ij}-\kappa_{k}\right)}_{+}^{2}+\sum_{k=1}^{K}b_{k}^{{\mathrm{CTR}}_{i}}{\left(t_{ij}-\kappa_{k}\right)}_{+}^{2}$
(1.5) Separate smoothing and distinct smoothing parameters for OSA and Control profiles	Same as structure (1.4) but differing variances for smoothing coefficients: $b_{k}^{{\mathrm{OSA}}_{i}}\sim N\left(0,\sigma_{b_{k}^{\text{OSA}}}^{2}\right)$ and $b_{k}^{{\mathrm{CTR}}_{i}}\sim N\left(0,\sigma_{b_{k}^{\text{CTR}}}^{2}\right)$

* The term OSA*_i_* indicates whether subject belongs to the OSA or Control group (1=OSA, 0=Control); similarly, *CTR_i_* indicates group membership for spline-based differences (1=Control, 0=otherwise).

**Table 2:** Semiparametric mixed model selection applied to twenty-four hour diastolic blood pressure.

	Model*				
	(1.1)	(1.2)	(1.3)	(1.4)	(1.5)
−2LL	−10065.38	−10066.22	−10085.25	−10061.73	−8897.343
AIC	−10045.38	−10044.22	−10059.25	−10035.73	−8869.343
*BIC*	−*9974.026*	−*9965.724*	−*9966.49*	−*9942.971*	−*8769.444*
*E_P_*	10.1143	11.1150	13.1204	18.1296	6.0343
AIC_adj_	−10045.15	−10043.99	−10059.01	−10025.47	−8885.274

* Results were obtained prior to covariance model selection and assume subject-specific random intercepts. Demographic and clinical characteristics included as covariates.

**Table 3:** Covariance model selection for the semiparametric mixed model (1.3) applied to twenty-four hour diastolic blood pressure.

	Covariance Model_*_		
	Random Intercepts	Exponential	Gaussian
−2LL	−10085.3	−11176.3	−11108.5
AIC	−10059.3	−11146.3	−11078.5
*E_P_*	10.3214	13.0746	12.9565
AIC_adj_	−10064.66	−11150.15	−11082.59

* Results were obtained for Model (1.3), which assumes mean response functions for groups differ by quadratic trend. Demographic and clinical characteristics included as covariates.

## Abbreviations

AIC: Akaike’s Information Criterion
BARS: Bayesian Adaptive Regression Splines
DBP: Diastolic blood pressure
LMM: Linear Mixed Model
LRT: Likelihood Ratio Test
ML: Maximum Likelihood
OI: Obstructive Index
OSA: Obstructive Sleep Apnea
REML: Restricted Maximum Likelihood
